# Pathogen-regulated genes in wheat isogenic lines differing in resistance to brown rust *Puccinia triticina*

**DOI:** 10.1186/s12864-015-1932-3

**Published:** 2015-10-05

**Authors:** Marta Dmochowska-Boguta, Sylwia Alaba, Yuliya Yanushevska, Urszula Piechota, Elzbieta Lasota, Anna Nadolska-Orczyk, Wojciech M. Karlowski, Waclaw Orczyk

**Affiliations:** Department of Genetic Engineering, Plant Breeding and Acclimatization, Institute – National Research Institute, Radzikow, 05-870 Blonie Poland; Department of Computational Biology, Institute of Molecular Biology and Biotechnology, Faculty of Biology, Adam Mickiewicz University, Umultowska 89, 61-614 Poznan, Poland; Department of Functional Genomics, Plant Breeding and Acclimatization, Institute – National Research Institute, Radzikow 05-870 Blonie, Poland

**Keywords:** Bread wheat, 14-3-3 protein, Calcium-mediated signaling protein, *Lr* gene, Pathogen-induced signaling, Plant-pathogen interaction, Serine/threonine protein kinase SSH, Transcriptome, *Triticum aestivum*, Wall-associated kinase

## Abstract

**Background:**

Inoculation of wheat plants with *Puccinia triticina* (*Pt*) spores activates a wide range of host responses. Compatible *Pt* interaction with susceptible Thatcher plants supports all stages of the pathogen life cycle. Incompatible interaction with Tc*Lr9* activates defense responses including oxidative burst and micronecrotic reactions associated with the pathogen’s infection structures and leads to complete termination of pathogen development. These two contrasting host-pathogen interactions were a foundation for transcriptome analysis of incompatible wheat-*Pt* interaction.

**Methods:**

A suppression subtractive hybridization (SSH) library was constructed using cDNA from pathogen-inoculated susceptible Thatcher and resistant TcLr9 isogenic lines. cDNA represented steps of wheat-brown rust interactions: spore germination, haustorium mother cell (HMC) formation and micronecrotic reactions. All ESTs were clustered and validated by similarity search to wheat genome using BLASTn and sim4db tools. qRT-PCR was used to determine transcript levels of selected ESTs after inoculation in both lines.

**Results and discussion:**

Out of 793 isolated cDNA clones, 183 were classified into 152 contigs. 89 cDNA clones and encoded proteins were functionally annotated and assigned to 5 Gene Ontology categories: catalytic activity 48 clones (54 %), binding 32 clones (36 %), transporter activity 6 clones (7 %), structural molecule activity 2 clones (2 %) and molecular transducer activity 1 clone (1 %).

Detailed expression profiles of 8 selected clones were analyzed using the same plant-pathogen system. The strongest induction after pathogen infection and the biggest differences between resistant and susceptible interactions were detected for clones encoding wall-associated kinase (GenBank accession number JG969003), receptor with leucine-rich repeat domain (JG968955), putative serine/threonine protein kinase (JG968944), calcium-mediated signaling protein (JG968925) and 14-3-3 protein (JG968969).

**Conclusions:**

The SSH library represents transcripts regulated by pathogen infection during compatible and incompatible interactions of wheat with *P. triticina*. Annotation of selected clones confirms their putative roles in successive steps of plant-pathogen interactions. The transcripts can be categorized as defense-related due to their involvement in either basal defense or resistance through an R-gene mediated reaction. The possible involvement of selected clones in pathogen recognition and pathogen-induced signaling as well as resistance mechanisms such as cell wall enforcement, oxidative burst and micronecrotic reactions is discussed.

**Electronic supplementary material:**

The online version of this article (doi:10.1186/s12864-015-1932-3) contains supplementary material, which is available to authorized users.

## Background

Brown rust caused by *Puccinia triticina* (*Pt*) is one of the most devastating wheat diseases, responsible for significant yield loses [1]. Resistance breeding is the best economic and ecological approach to control plant diseases. To date, 71 *Lr* loci conferring resistance to *Puccinia triticina Pt* have been identified in wheat since characterization of the first rust resistance gene in 1946 [2]. Roughly half of them have been found in common hexaploid wheat, while the remaining ones were introgressed from donor species by means of interspecific hybridization (http://www.ars.usda.gov/SP2UserFiles/ad_hoc/36400500Resistancegenes/Lrgene.xls).

Most of the *Lr* genes have been bred, as a single gene or in combination with others, into wheat to obtain resistant cultivars. Despite the importance of resistance breeding, information about the vast majority of *Lr* genes is derived mostly from plant phenotype analyses. So far, only a small fraction of *Lr* genes have been cloned and characterized. The list includes four genes: *Lr1*, *Lr10*, *Lr21* and *Lr34*. The *Lr1* gene, located at the distal end of the long arm of chromosome 5D, is part of a large gene family [3]. In contrast, *Lr10* is a single copy gene located on wheat chromosome 1AS and confers enhanced resistance to brown rust [4]. *Lr21* is located in a gene-rich region of the wheat 1DS chromosome [5]. The three genes, conferring so-called seedling resistance, encode proteins with coiled coil (CC), nucleotide-binding site and leucine-rich repeat (NBS-LRR) domains. Despite similar protein architecture, they show a low (18–21 %) level of amino acid sequence similarity [3]. The *Lr34* gene confers adult plant resistance (APR), which remained unbroken by the pathogen for over 50 years. Lr34 protein contains an ATP binding cassette (ABC) domain similar to ABC transporters which are also known as multidrug resistance (MDR) [6]. The ABC protein encoded by *Lr34* and the NBS-LRR proteins encoded by *Lr1*, *Lr10* and *Lr21* are significantly different considering their architecture and function. It is highly probable that the resistance they provide relies on very different mechanisms. Characterization of only four *Lr* genes indicates that the resistance against *Pt* relies on diverse biological mechanisms. Cloning of other genes should widen the range of mechanisms known to be involved in plant resistance. This notion is consistent with the diversity in plant-pathogen interactions as well as different effectiveness and durability of resistance provided by various *Lr* genes and gene combinations [7].

The *Lr9* gene represents an introgression from *Aegilops umbellulata* and is located on wheat chromosome 6BL [8]. It has been selected for this study because it confers highly efficient resistance, which for a long period remained unbroken by the pathogen. Although there are reports on *Pt* isolates virulent to *Lr9*, the gene is still considered as an important component of efficient brown rust resistance [9]. Analysis of host-pathogen interaction shows that both susceptible Thatcher and resistant Tc*Lr9* plants support the first steps of *Pt* development: i.e. germination of urediniospores, formation of appressoria and development of haustorium mother cells (HMC). Suppression of *Pt* in Tc*Lr9* begins after formation of haustoria, although the actual recognition takes place shortly after appressoria formation; therefore the resistance is referred to as post-haustorial [10]. In the susceptible Thatcher plants the pathogen life cycle is completed 7–8 days after inoculation, with formation of large uredinia filled with urediniospores. The resistant Tc*Lr9* shows no symptoms of rust infection and no uredinia are formed. Although histopathologically detectable differences between compatible and incompatible interactions are visible only after formation of HMCs 24–48 h post inoculation (hpi), the reactions of Tc*Lr9* leading to resistance start within a very short time after inoculation [10]. Genes participating in oxidative burst, i.e. peroxidases and NADPH oxidases, are highly induced already 8 hpi. Gene activation corresponds very well with outset of H_2_O_2_ accumulation. Micronecrotic reactions, not detected in Thatcher, were temporally and spatially associated with 87 % of HMCs in resistant Tc*Lr9*. Micronecrosis together with oxidative burst represent a strong and localized response leading to efficient inhibition of the pathogen growth and nearly symptomless resistance [10, 11].

Resistance *R* genes encode proteins responsible for recognition of pathogen/pathogen-associated molecules and lead to incompatible interaction. This requires coordinated regulation of a wide range of defense-responsive (*DR*) genes and defense processes. The *DR* genes can be experimentally identified by one of several methods: differential display [12], cDNA-AFLP [13], microarray analysis [14] or suppression subtractive hybridization SSH. The former approach proved to be fruitful in identifying transcripts regulated in diverse plant-pathogen systems: e.g. wheat infected with *Fusarium graminearum* [15], rice with *Xanthomonas oryzae* pv. *Oryzae* [16], barley inoculated with *Pyrenospora teres* [17] and olive roots colonized with endophytic *Pseudomonas fluorescens* [18]. The goal of the present study was to identify by the SSH approach transcripts specifically regulated during incompatible interaction of Tc*Lr9* plants with *P. triticina*.

## Methods

### Inoculation of wheat seedlings with brown rust and histopathological analysis

The cultivar Thatcher susceptible to brown rust and the isogenic line carrying the *Lr9* rust resistance gene (Tc*Lr9*) [19] were used as plant material for construction of the subtractive cDNA library. The isogenic lines – strongly resistant Tc*Lr26* and medium resistant Tc*Lr24* and Tc*Lr25* – were used for expression analysis of selected cDNA clones. A single spore isolate of *P. triticina* with an established avirulence⁄virulence formula (Additional file 1: Table S4) [10] was used for plant inoculation. The wheat seedlings were grown in a growth chamber at 22 °C with a 16 h photoperiod and illumination intensity of 60 μE m^−2^ s^−1^. To identify time points crucial in the wheat response to brown rust infection the primary leaves of 7-day-old Tc*Lr9* seedlings were inoculated using a soft brush with spores suspended in water with Tween 20 at a density of 1 mg ml^−1^ for microscopic observations and 20 mg ml^−1^ for RNA isolations. Calcofluor white staining and assessment of inoculation efficiency was done using 6 leaves randomly selected from a set of 30 inoculated seedlings. Final spore density on the leaf surface was in the range of 40–150 spores · cm^−2^ for microscopic observations. Spore density was assessed by counting 1000–2000 infection sites per leaf. Directly after inoculation, plants were incubated for 24 h in a dark growth chamber at 18 °C and 100 % humidity, and then cultivated in the same conditions as seedlings prior to inoculation. Leaf samples of Tc*Lr9*, collected 4, 8, 11, 16, 20, 24, 28, 32, 36 and 44 h post inoculation (hpi), were stained with calcofluor white as described earlier [10]. Briefly, leaf samples were cleared and fixed in ethanol ⁄dichloromethane (3:1 v/v) with 0.15 % trichloroacetic acid for 24 h, washed twice in 50 % ethanol, twice in 0.05 M sodium hydroxide, three times in water, once in 0.1 M Tris–HCl, pH 8.5, and then stained in 3.5 mg ml^−1^ of calcofluor white dissolved in 0.1 M Tris–HCl, pH 9, and washed in water. The stained samples were examined under a fluorescence microscope (Nikon Diaphot, epifluorescence optics with excitation 340–380 nm, barrier filter 420 nm and dichroic mirror 400 nm). These conditions allowed the visualization of pathogen development, i.e. germinating spores, appressoria and haustorium mother cells (HMC). This procedure also allowed detection of the host cell micronecrosis autofluorescence adjacent to HMC. The number of germinating spores, spores forming appressoria, spores forming HMC and the number of necrotic spots with autofluorescence were counted on the whole area of a randomly selected leaf. Results were expressed as the ratio of spores forming appressoria, spores forming HMC and the ratio of necrotic spots to all germinating spores. The upper part of each leaf (1.5 cm) collected for RNA isolation was stained with calcofluor white to check the efficiency of rust inoculation. Low inoculum density (40–150 spores · cm^−2^) facilitating good microscopic observations was applied for leaves used only for histopathological analyses. High inoculum density (described below) was applied for leaves used for RNA extraction.

### SSH library construction and differential screening

To identify wheat genes involved in the response to pathogen infection, leaf samples were collected from the susceptible Thatcher and resistant Tc*Lr9* line 12, 20, 26, 32 and 44 hpi. The selected time points represented the most significant plant-pathogen interaction events: 12 hpi marked the presence of appressoria in almost all germinating spores, 20 and 26 hpi the outset of HMC formation and micronecrotic reaction respectively, 32 hpi highlighted the progressive development of all the structures, i.e. appressoria, HMC and micronecrosis, and the final 44 hpi time marked the presence of all the above structures in a well-developed plant-pathogen interaction. To reduce the heterogeneity of the samples the RNA was isolated only from leaves with a high density of germinating spores. This was assessed by calcofluor white staining and spore counting on the middle part of each leaf collected for RNA extraction. Only leaves with over 90 % of stomata accompanied by rust appressoria were used as plant material for RNA extraction. The samples taken from 4 selected leaves from each time point after inoculation were pooled, ground in liquid nitrogen and subjected to RNA extraction using the AGPC (acid guanidinium thiocyanate-phenol-chloroform) method according to a modified procedure described previously [20]. The RNA concentration and the A_260_/A_280_ ratio were measured with NanoDrop ND-1000 (NanoDrop Technologies, USA). The quality of total RNA was assessed using agarose gel electrophoresis. 5 μg of the total RNA was incubated with 2 U of DNase (DNase I recombinant RNase-free, Roche, USA) for 15 min at 37 °C. DNase was inactivated, according to the manufacturer's protocol by addition of EDTA and heating at 75 °C for 10 min. The lack of genomic DNA was verified by PCR amplification using 200 ng of DNase digested RNA as a template and PB3/PB4 primers (Additional file 2: Table S1) designed for the wheat *pinB* gene [21]. RNA samples giving no detectable *pinB* product on agarose gel after 40 cycles of amplification were used for further steps of the procedure.

The DNase digested total RNA extracted from leaves 12, 20, 26, 32 and 44 hpi were pooled in equal amounts and used for isolation of poly(A)^+^RNA with Oligotex Suspension (Qiagen, USA) according to the manufacturer’s protocol. Two poly(A)^+^RNA samples obtained from the susceptible cv. Thatcher (driver) and from the resistant Tc*Lr9* (tester) were used for SSH library construction. The library was constructed using the BD PCR-Select cDNA Subtractive Hybridization Kit (Clontech, USA) according to the manufacturer’s protocol. After tester cDNA digestion with *Rsa*I and ligation to adaptor 1 and 2R, two rounds of hybridization and PCR amplification were performed to enrich the differentially expressed sequences. The products of the second round of PCR of subtracted cDNA were cloned into pGEM-T Easy Vector (Promega, USA) using the T/A cloning approach and transformed into *Escherichia coli* DH5α. All *E. coli* colonies potentially carrying an insert-containing vector were individually selected based on the blue/white screening. PCR was performed with 1 μl of an overnight bacterial culture as a template and a pair of universal primers, T7/SP6 (Additional file 2: Table S1), flanking the cDNA insert. The PCR products were evaluated in 1.2 % agarose gel electrophoresis to analyze the length of the insert of each clone. Colonies with a confirmed cDNA insert were used to isolate plasmid DNA for differential screening.

Differential screening was performed with the PCR-Select Differential Screening Kit (Clontech, USA) according to the manufacturer’s instructions. All cDNA inserts representing individual SSH clones were amplified using isolated plasmid DNA as a template, Nested PCR Primer1 and Nested PCR Primer2R from the BD PCR-Select cDNA Subtractive Hybridization Kit (Clontech, USA) and Taq Platinum DNA Polymerase (Invitrogen, USA). The size and the quantity of the amplification products were examined in agarose electrophoresis. The 100 ng samples of the amplicons, the negative controls of the wheat histone EST and H_2_O as a blank control were mixed with an equal volume of NaOH 0.6 N for alkali denaturation. The samples were manually spotted with a multi-channel pipette on nylon membranes, neutralized with Tris · HCl 0.5 M (pH 7.5) 2 min, washed with H_2_O and immobilized for 30 min at 120 °C.

The probe for the differential screening was obtained and quantified using the PCR DIG Probe Synthesis Kit (Roche, USA) according to the manufacturer’s protocol. The 50 μl synthesis and labeling PCR reaction mix contained 5 μl of 10x PCR Reaction Buffer, 2 μl of nested PCR primer1 10 μM, 2 μl of nested PCR primer2R (Additional file 2: Table S1) 10 μM, 1 μl of 10x PCR DIG Mix, 34.25 μl of H_2_O, 0.75 μl of enzyme mix and 1 μl of the template containing 20 ng of cDNA obtained after subtractive hybridization. After the initial denaturation at 94 °C for 2 min, 30 cycles of amplification were carried out at 94 °C for 30 s, 68 °C for 30 s, and 72 °C for 40 s, followed by the terminal extension step at 72 °C for 7 min. The serial dilutions of the probe and the labeled control DNA provided by the manufacturer were loaded on the nylon membrane and immobilized for 30 min at 120 °C. Detection was carried out according to the manufacturer’s protocol. The membranes were exposed to X-ray film (X-Omat AR, Kodak, USA) for 10–60 min. The amount of the labeled probe was estimated based on the known amount of control DNA. The membrane with the spotted cDNA was hybridized in stringent conditions. It was pre-hybridized with 20 ml of the DIG Easy Hyb (Roche, USA) at 42 °C. After 30 min the solution was replaced with the hybridization buffer containing the labeled and heat denatured probe. Hybridization was carried out for 20 h at 42 °C and was followed by washing with 2x SSC, 0.1 % SDS for 5 min in RT, twice with 0.5x SSC, 0.1 % SDS for 15 min at 68 °C and 5 min with washing buffer. DIG detection was done directly after the last washing. The membrane was incubated for 2 min in 100 ml of maleic acid buffer, 30 min in 1x blocking solution (Roche, USA), 30 min in 20 ml of Anti-Digoxigenin-AP Conjugate dissolved in 1x blocking solution and washed with washing buffer (Roche, USA). Unbound antibodies were removed by 2 min washing in detection buffer and the membrane was evenly covered with 1 ml of CSPD diluted 1/100 v/v in detection buffer. After 5 min incubation at 37 °C the membrane was exposed to an X-ray film (X-Omat AR, Kodak, USA) for 10–60 min depending on the strength of the chemiluminescent signals. The results from the hybridizations were recorded for each clone, and those showing the most marked differential expression were selected for sequencing.

### Sequencing of EST clones

Sequencing was carried out by the Laboratory of DNA Sequencing and Oligonucleotide Synthesis, IBB PAS using SP6 and T7 primers. The edited sequences devoid of the primer sequence and the sequencing ambiguities were used to query the GenBank database at NCBI http://www.ncbi.nlm.nih.gov using the BLAST sequence comparison algorithms. The sequences of the wheat cDNA clones identified in this work were deposited in GenBank http://www.ncbi.nlm.nih.gov/Genbank (Additional file 3: Table S2).

### Assembly of EST contigs, quality screening and functional annotation

To remove redundancy and to extend the sequence span the identified EST sequences were used for contig (unique transcripts) assembly along with transcriptomic data (EST, cDNA) from GenBank with the CAP3 program [22]. All EST clusters were validated by similarity search to wheat genome using BLASTn and sim4db tools [23, 24]. Transcripts showing at least 90 % coverage and 80 % similarity were considered as originating from wheat. Remaining, non-mapping sequences were compared to fungi genomic databases (NCBI nt fungi, JGI MycoCosm and Ensembl Fungi) using provided BLAST-based search tools [25, 26]. In the next step, all sequence clusters were used for functional annotation. ESTscan [27] was applied for estimation of transcript protein coding potential. For algorithm training data from *Brachypodium distachion* (dbEST: 128092; UniGene: 10698; RefSeq: 24640) were downloaded from NCBI databases. The predicted wheat peptides were subsequently mapped to the NCBI nr protein database using BLASTp with default parameters [28]. Additionally, all wheat transcripts were mapped to Rfam v10.1 and RepBase v17.04 databases using BLASTn to identify known non-coding RNA sequences and TE derived transcripts, respectively [29, 30]. Assignment of GO terms was based on the ‘molecular function’ category, because it represents the most detailed information for annotation purposes. The GO terms were extracted from the gene association (GOA) UniProt file, downloaded from the Gene Ontology FTP site [ftp://ftp.ebi.ac.uk/pub/databases/GO/goa/] using the in house developed Python script. In cases where no GO terms could be found in the GOA file, the classification information was manually downloaded from organism-specific databases (e.g. TAIR and Gramene [ftp://ftp.arabidopsis.org/home/tair/Ontologies/Gene_Ontology/, ftp://ftp.gramene.org/pub/gramene/CURRENT_RELEASE/data/ontology/go/]. The CateGOrizer tool was used for final reduction of GO terms complexity along with the Plant GO Slim dataset available from the CateGOrizer web [31]. The HMMER3 program with profiles downloaded from Pfam v26.0 [32, 33] was used for prediction of functional protein domains with the e-value parameter set to 0.001. ESTs with no detectable protein coding potential were scanned for repetitive sequences using the BLASTn program. Repetitive sequences were downloaded from the Wheat Genome Database [34] and Repbase v18.09 repository [30]. Alignments covering at least 40 % of the EST sequence with more than 70 % identity were considered as TE-related. Transcripts without assigned function were tested for sRNA coding potential. The microRNA sequences from miRBase v18.0 and *Triticum aestivum* sRNA-Seq data from the GEO database (GSE36867, GSE32476, GSE27327, GSE22048 and GSE16177) were mapped onto assembled EST transcripts with the BLASTn program using parameters adjusted for short sequences (−word_size 6 -gapopen 3 -penalty −1) [35, 36]. Only sequences originating from a forward strand without mismatches were considered.

### Quantitative RT-PCR

Quantitative RT-PCR was used to determine transcript levels of selected ESTs at the particular time point after inoculation in both isogenic lines. The leaves collected 0, 8, 16, 24, 32 hpi and 2, 3, 4 and 5 days post inoculation (dpi) were stained with calcofluor white to verify high density of germinating spores. RNA extraction, DNase digestion, and checking RNA purity from genomic DNA were done as for SSH library construction. Leaf RNA extracted from 3 independently inoculated seedlings represented 3 biological repetitions of expression profiling. The reverse transcription reaction was performed using the Revert Aid First Strand cDNA Synthesis Kit (Fermentas, Lithuania) according to the manufacturer’s protocol. The cDNA samples were diluted 20x and directly used as templates for qPCR.

The standard qPCR reaction mix was composed of: 5 μl of the mastermix (2x Sso-Fast Eva Green Supermix, Bio-Rad Laboratories, Hercules, USA), 0.3 μl of primer F (10 μM), 0.3 μl of primer R (10 μM), 1 μl of cDNA and 3.4 μl of water. The reactions were performed in a Corbett Rotor-Gene 6000 5-plex thermocycler in conditions summarized in Additional file 2: Table S1. A melting curve analysis (72 °C to 95 °C) was performed to ensure the specificity of the amplification. The size of each amplicon was verified by gel electrophoresis. Transcript quantitation in each analyzed combination of isogenic line and the time point was done in three independent biological replications and each reaction was run in three technical repetitions. Concentrations ranging from 10^5^ to 10^11^ copies of analyzed amplicon per reaction were used as the standards for 18S and 10^2^–10^8^ copies per reaction for other genes. Reaction efficiencies (RE) were between 0.8 and 1.1. Relative expression of the analyzed gene was calculated by two standard curves method with 18S rRNA as a reference using Rotor-Gene 6000 software v 1.7. For statistical comparison ANOVA followed by Tukey’s post-hoc test was used (STATISTICA 10, StatSoft). Only *p* values <0.05 were considered as statistically significant.

### CVTR

In order to compare the possible impact of the expression of each analyzed gene on host-plant interaction the cumulative value of transcript rate (CVTR) was used as described previously [11]. The CVTR denoted the total transcript accumulation rate during 5 days of infection and was calculated as the definite integral of the function of the accumulation rate between 0 and 5 dpi. The calculated value represents the area below the graph of the accumulation rate delimited by time 0 and 5 dpi for each gene. Since the CVTR takes into account both the transcript accumulation rate and the duration of expression, it allows for a direct comparison of all genes in tested lines. This enables the evaluation of the impact of genes’ expression on the analyzed process. The Spearman correlation coefficient was calculated (using Statistica version 10, StatSoft) between the ranked variables of the CVTRs of the analyzed SSH clone in susceptible Thatcher, medium resistant (Tc*Lr24*, Tc*Lr25*) and highly resistant (Tc*Lr9*, Tc*Lr26*) lines.

## Results

### Determination of time points crucial for plant-pathogen interaction

**Fig. 1:**
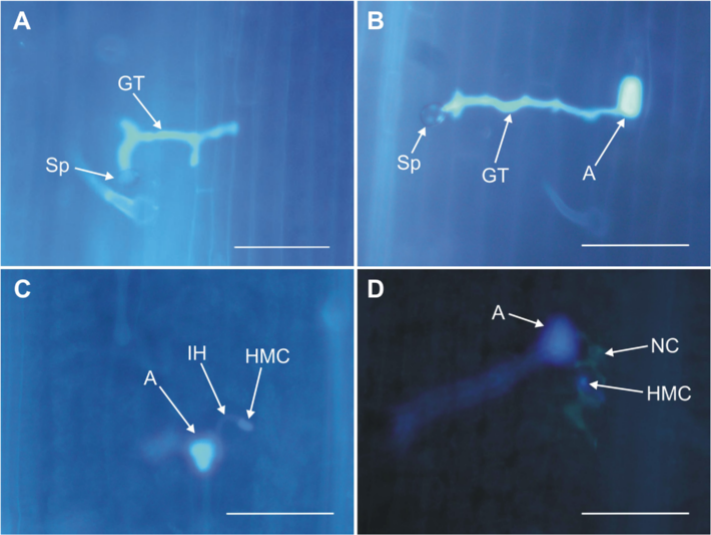
Microscopic visualization of brown rust (*P. triticina*) spore development and micronecrotic reaction of plant cells adjacent to haustorium mother cell (HMC) in Tc*Lr9*. Leaves of 7-day-old seedlings were inoculated with brown rust spores. Leaf samples, collected 4, 8, 11, 16, 20, 24, 28, 32, 36 and 44 h post inoculation (hpi), were stained with calcofluor white and analyzed under a fluorescence microscope. Representative images are shown with germinating spore, 4 hpi (**a**), appressorium on the guard cells, 4 hpi (**b**), haustorium mother cell, 16 hpi (**c**), necrotic cells formed near haustorium mother cell, 28 hpi (**d**). Indicated are: spore of brown rust (Sp), germ tube of a germinating spore (GT), appressorium (**a**), infection hypha (IH), haustorium mother cell (HMC), and necrotic cells (NC). Bar = 100 μm

To identify time points crucial in the plant response to pathogen infection, seedlings of the resistant line Tc*Lr9* were inoculated with brown rust spores. The leaf samples, collected 0, 4, 8, 11, 16, 20, 24, 28, 32, 36 and 44 hpi were used for histopathological analysis (Fig. 1). The percentage of germinating spores that formed appressoria and haustorium mother cells (HMCs), as well as the percentage of infection sites with micronecrotic reactions, was scored.

**Fig. 2:**
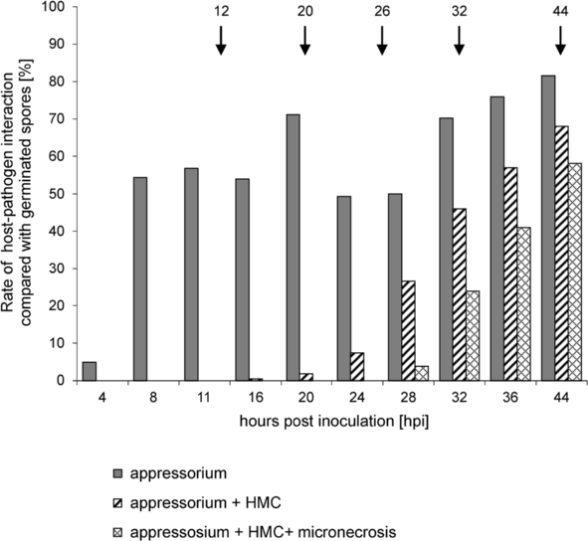
Profiles of *P. triticina* development (appressoria, HMCs) and rates of micronecrotic responses of Tc*Lr9* plants. Shown are: i) the percentage of germinating spores which formed appressoria, ii) the percentage of germinating spores with appressoria and HMCs (appressorium + HMC) and iii) the percentage of spores with appressoria, HMC and micronecrosis. Arrows indicate the time points selected for RNA extraction and SSH library construction (12, 20, 26, 32 and 44 hpi)

The fraction of spores forming appressoria was 4.9 % at 4 hpi and 54.4 % at 8 hpi. Between 11 and 44 hpi the value ranged from 56.8 % to 81.6 %. The first haustorium mother cells (HMCs) were detected 16 hpi. The portion of spores with HMCs at 16 hpi was 0.4 % and this value steadily increased to 68 % at 44 hpi. Necrotic cells adjacent to HMCs, which indicated a plant response to the pathogen infection, were detected at 28 hpi, and the percentage of spores inducing a necrotic reaction was 3.8 %. This value was 23.9 % at 32 hpi and increased to 58.1 % at 44 hpi (Fig. 2).

Total RNA for SSH library construction was extracted from leaves collected 12, 20, 26, 32 and 44 hpi. The selected time points represent the most important steps of plant-pathogen interaction. The first one (12 hpi) represented the high percentage of germinating spores, shortly before formation of HMCs. The time point 20 hpi represented the beginning of HMC formation, and 26 hpi marked the high ratio of HMCs, shortly before the necrotic reactions. Progressive development of micronecrotic reactions, i.e. the plant response, closely associated with formation of HMCs, was significant at 32 hpi. The high ratio of HMCs and strong necrotic response were the most important features at 44 hpi (Fig. 2).

### SSH library construction and identification of differentially expressed clones

**Fig. 3:**
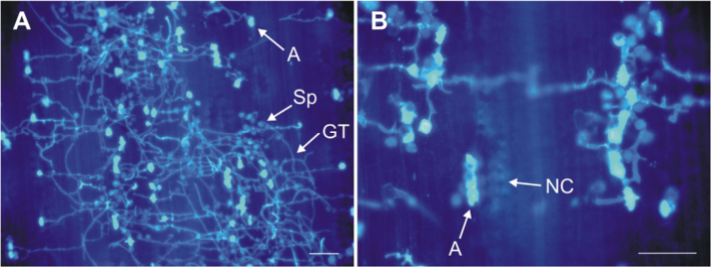
Microscopic picture of leaf samples with high density of germinating *P. triticina* urediniospores (**a**), higher magnification of high density urediniospores with visible necrotic cells (**b**). Indicated are: spore of brown rust (Sp), germ tube of a germinating spore (GT), appressorium (A), necrotic cells (NC). Bars = 100 μm

The uniform inoculation of both isogenic lines was confirmed by microscopic observation of germinating spores in leaf samples stained with calcofluor white. To ensure proper pathogen-induced gene expression in analyzed samples, total RNA was isolated from leaves with confirmed high density of germinating spores (results not included). Only leaves where 70-90 % of guard cells were occupied by appressoria were used for RNA isolation and for further analysis (Fig. 3).

**Fig. 4:**
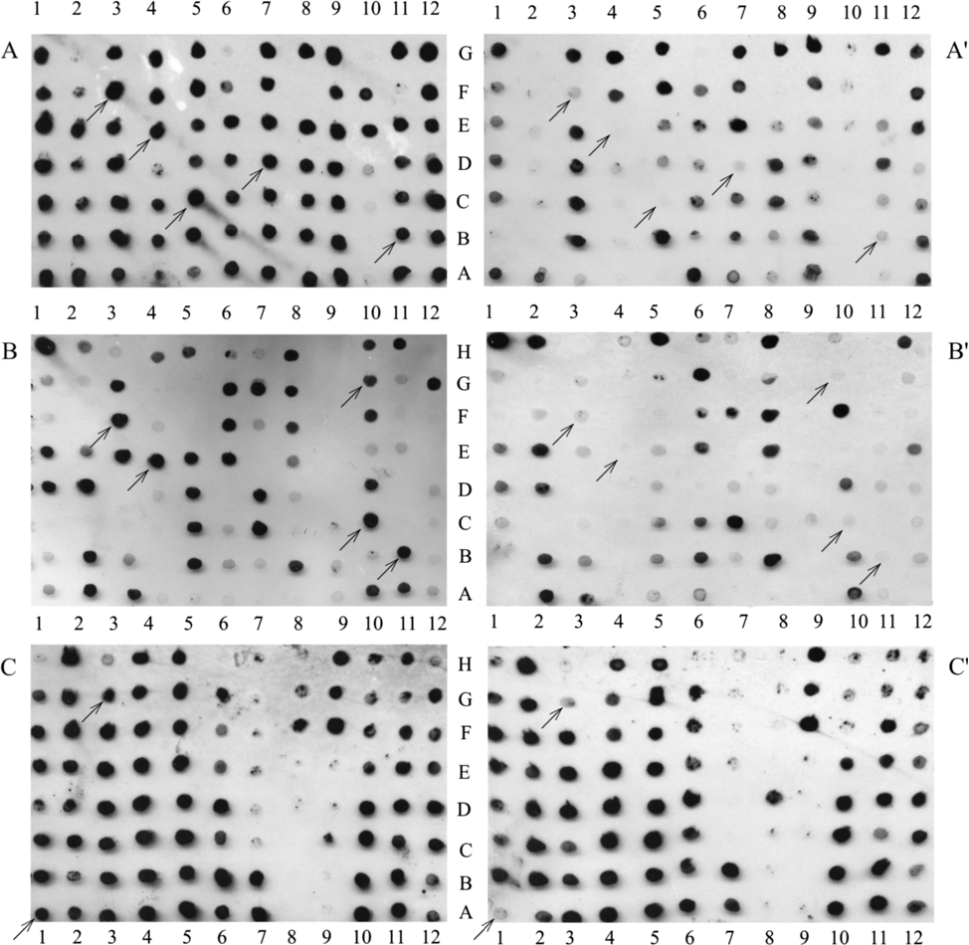
Autoradiogram of differential screening. Macroarrays of cDNA spots hybridized with DIG-labeled forward subtracted cDNA (left panel A, B, C) and reverse subtracted cDNA (right panel A', B', C'). Indicated are examples of the EST clones regulated during brown rust infection and selected for sequencing and further analysis

In total 793 cDNA clones were isolated and subjected to differential screening by hybridization with DIG-labeled probes (Fig. 4). 247 EST clones represented by the most marked differential expression (see examples shown in Fig. 4) were selected for sequencing and further analysis. After initial evaluation and quality screening, 183 selected clones were deposited in GenBank http://www.ncbi.nlm.nih.gov/Genbank (Additional file 3: Table S2).

### Gross functional annotation of EST tags

**Fig. 5:**
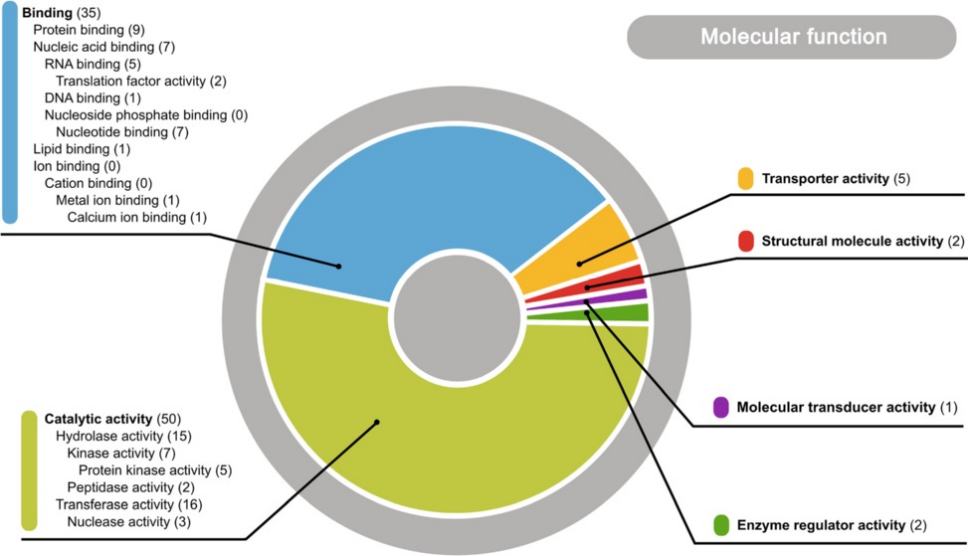
Functional annotation of predicted proteins based on Gene Ontology classification. Only main GO term categories from molecular function domain are presented on the chart. Lower level terms are shown as part of the section’s description. Numbers in brackets represent sequences annotated with listed feature

The EST sequences were mapped to the wheat genome and available fungal sequences to detect putative contaminations. Only one sequence (JK818344_JK818346) could not be mapped to any reference dataset and it did not reveal any significant similarity to publicly available sequences. Two sequences (JK818343 and JK818359) show strong similarity to the *P. triticina* genomic sequence and represent differentially expressed pathogen transcripts. No function could be assigned to them based on conserved domain screening and similarity searches (see later). 183 EST clones were assembled into 152 unique transcripts (contigs; see Materials & Methods). 113 out of 152 clustered EST sequences show protein coding potential when analyzed with ESTscan trained on *Brachypodium distachyon* sequences. The average predicted protein length was 135 aa, with maximal and minimal size of 271 and 34 aa, respectively. Additionally, 16 contigs were classified as protein-coding based on a BLASTx search. In total 129 out of 152 sequences (85 %) were predicted to encode proteins. Analysis of the protein sequences with the Pfam database for presence of functional domains allowed the identification of at least one motif in 82 sequences. Among them 3 clones (JK818321_JK818338, JG969002_JG968938_JG968937, JG968929) show complex domain architecture with 5 different functional amino acid motifs. The Gene Ontology classification system was used to annotate 89 of the tested proteins (Fig. 5). Among them the most represented categories were ‘catalytic activity’ and ‘binding’. The predicted molecular activities included ‘oxidation-reduction processes’, ‘phosphorylation’, ‘transport processes’, ‘protein folding’ as well as ‘responses to stress’ and ‘regulation of transcription and translation’. 11 sequences were predicted to be localized in plastids and 24 show properties characteristic for membrane proteins.

The remaining, non-protein coding transcripts were analyzed with mature miRNA data from miRBase. Only one fragment (JG968965) showed weak (three mismatches) similarity to gma-miR862b. The next-generation sequencing (NGS) data (GEO accession numbers: GSE36867, GSE32476, GSE29243, GSE27327, GSE22048 and GSE16177) did not confirm the presence of any miRNA-like molecule originating from this transcript.

Further analysis with sRNA sequencing data resulted in the identification of three additional transcripts with putative miRNA coding potential. JK818366 displayed a short read mapping pattern resembling the miR/miR* duplex. However, this sequence fails to fold into a canonical hairpin structure. Two other EST sequence clones (JK818342 and JK818383) form proper hair-pin structure but their sRNA coding capacity could not be unambiguously confirmed in NGS sequencing data. Using the plant repeated sequence resources (see Methods), we were able to identify one of the contigs as a transcript derived from a transposable element. The JK818394 sequence shows the highest similarity to the Gypsy-3-LTR family and encodes a putative 138 amino acid (aa) peptide. Interestingly, the predicted aa sequence does not display any similarity to known proteins, with the exception of a 26 aa fragment, which is highly similar to calcium/calmodulin-dependent serine/threonine-protein kinase 1 from *Triticum urartu*. Combined results of gross functional annotation are presented in Additional file 4: Table S3.

### Modeling the roles of differently expressed genes during the pathogen response

**Fig. 6:**
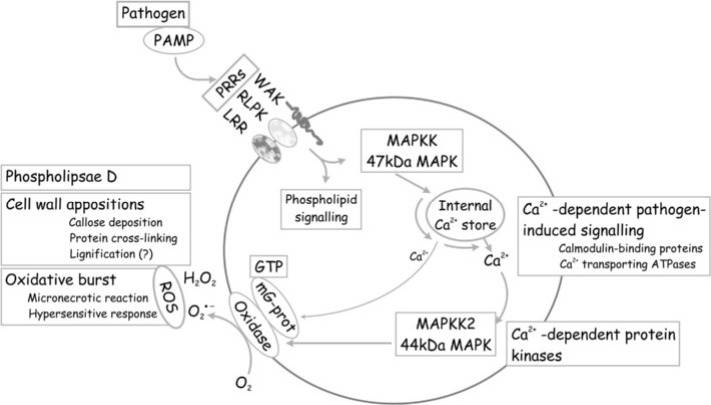
Schematic representation of pathogen-induced signal transduction pathways. The indicated components of the pathway are: pathogen associated molecular pattern PAMP (pathogen-derived elicitor), pattern recognition receptors PPR, MAP kinases and protein kinases, components of calcium-dependent pathogen-induced signaling and components of oxidative burst

Pathogen infection recognized by host receptors initiates a signaling pathway and triggers a complex host response. The cDNA clones identified in the wheat subtractive library represent genes involved in different phases of the plant-pathogen interaction (Fig. 6). Plant recognition of pathogen infection depends on interaction of host receptors with pathogen-derived or pathogen-induced elicitors. This step, necessary for triggering the host response, is mediated by receptor-like protein kinases (RLKs), proteins with nucleotide-binding site–leucine-rich repeat (NBS-LRR) domains and wall-associated kinases (WAKs). Twenty identified clones represent a putative protein kinase including mitogen-activated protein MAP kinase and five clones represent a putative receptor-like wall-associated kinase or receptor with an LRR domain.

Twelve clones represent transcripts involved in oxidation reduction processes. The putative respiratory burst oxidase homologue *Rboh* (JG968934) encodes a crucial enzyme responsible for generation of reactive oxygen species (ROS) during pathogen-induced oxidative burst. Clone JK818365 represents an MAP kinase responding to hydrogen peroxide and possibly participating in ROS-dependent signal transduction.

A group of 12 clones represent transcripts involved in calcium-dependent signaling. This group includes cluster JG968933_JK818362 with Ca^2+^ and ATP-binding activity and a cell wall localization domain. Clones possibly involved in pathogen-induced phospholipid signaling are represented by 4 clones. Clone JG968941 encodes callose synthase, a key enzyme necessary for pathogen-induced callose deposition and cell wall enforcement.

### Expression profiles of selected clones

**Fig. 7:**
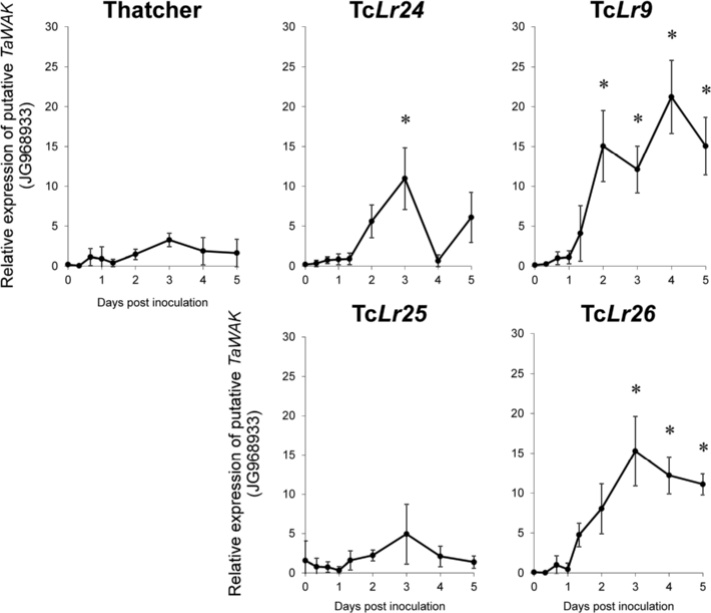
Expression profile of the putative wall-associated kinase *TaWAK* (JG968933) in susceptible cultivar Thatcher, medium resistant (Tc*Lr24*, Tc*Lr25*) and highly resistant (Tc*Lr9*, Tc*Lr26*) isogenic lines. Data represent mean value of the relative transcript accumulation rate with standard deviation between the three independent biological repetitions. * - cumulative *p* < 0.05 by analysis of variance (ANOVA) and *p* < 0.05 Tuckey’s test of *Lr* lines compared with Thatcher

**Fig. 8:**
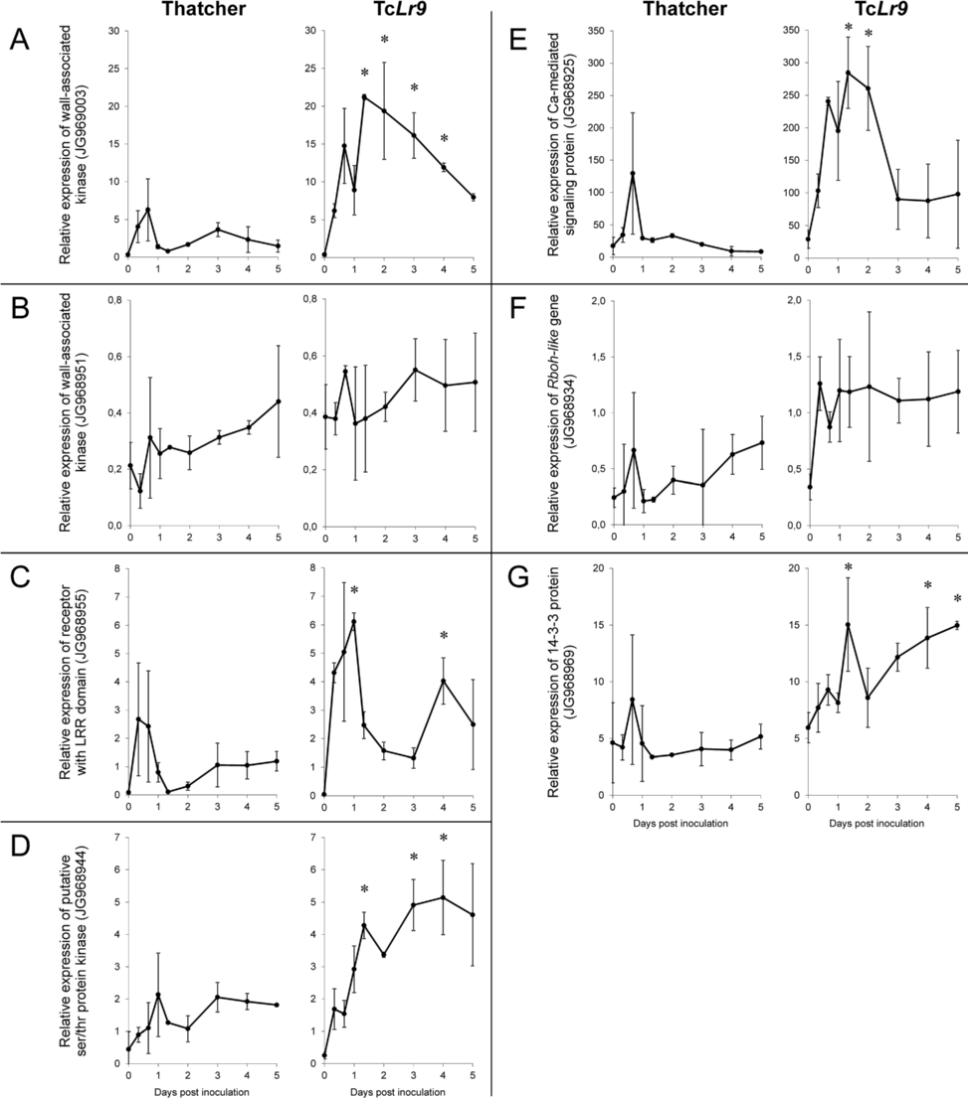
Expression profiles of selected clones in susceptible Thatcher and resistant Tc*Lr9*: wall-associated kinases (WAK) JG969003 and JG968951 (**a** and **b**), receptor with leucine reach repeat (LRR) domain JG968955 (**c**), putative serine/threonine protein kinase JG968944 (**d**), calcium-mediated signaling protein JG968925 (**e**), Rboh-like gene JG968934 (**f**) and 14-3-3 protein JG968969 (**g**). Data represent mean value of the relative transcript accumulation rate with standard deviation between the three independent biological repetitions. * - cumulative *p* < 0.05 by analysis of variance (ANOVA) and *p* < 0.05 Tuckey’s test of Tc*Lr*9 compared with Thatcher

Expression of the putative receptor like wall-associated kinase *TaWAK* (JG968933) is strongly induced in wheat inoculated with brown rust. The expression, much stronger in resistant lines (*Lr9*, *Lr24*, *Lr25* and *Lr26*) than in the susceptible Thatcher, clearly differentiates medium (Tc*Lr24*, Tc*Lr25*) and highly resistant (Tc*Lr9*, Tc*Lr26*) lines (Fig. 7). Wall-associated kinases are also represented by two other clones, JG969003_JG968950 and JG968951. Expression of both is pathogen dependent. The first one, JG969003, is transiently induced in inoculated leaves of susceptible Thatcher and strongly and stable activated in resistant Tc*Lr9* (Fig. 8a). The second, WAK encoding clone (JG968951) shows increasing expression in both Thatcher and the resistant line, although the differences between the two lines were not statistically significant (Fig. 8b). The transcript level in the plant-pathogen system is often site-specific and is localized in the cells surrounding the infection site. Thus the expression level depending on the actual inoculum density might vary in the biological repetitions. The results of our earlier gene profiling [11] indicate that this as well as the natural variability of two living and interacting organisms might result in relatively large standard errors at certain experimental time points.

The cumulative value of transcript rate (CVTR) was introduced to compare expression profiles of tested SSH clones and to evaluate their possible impact on plant-pathogen interaction in a particular line. The *TaWAK* (JG968933) CVTRs correlated with the resistance levels of the tested lines. The lowest value was established in susceptible Thatcher, intermediate were in medium resistant (Tc*Lr24*, Tc*Lr25*) and the highest were in highly resistant (Tc*Lr9*, Tc*Lr26*) lines. The Spearman correlation coefficient of the ranked resistance levels and the CVTRs of *TaWAK* (JG968933) was 0.947 and the significance level *p* = 0.014 (Table 1).

**Table 1:** Spearman correlation coefficient of ranked resistance levels and CVTRs of *TaWAK* clone JG968933

Isogenic lines	Group I	Group II		Group III	
Calculations	Susceptible	Medium resistant		Highly resistant	
	Thatcher	Tc*Lr24*	Tc*Lr25*	Tc*Lr9*	Tc*Lr26*
CVTR of *TaWAK* JG968933 (Fig. 7)	8.1	20.4	11.2	56.3	42.6
Spearman correlation coefficient	0.947				
Significance level	*p* = 0.014				

A putative receptor with a leucine-rich repeat (LRR) domain (JG968955) is strongly and transiently induced at 8–16 hpi in both susceptible and resistant plants. The first upregulation peak is followed by a second one 3–4 dpi. Although the expression pattern is similar in both lines, the relative transcript level in the resistant Tc*Lr9* is over twice as high as in the susceptible Thatcher (Fig. 8c). Expression of putative serine/threonine protein kinase (JG968944) in the susceptible Thatcher shows temporal induction 24 hpi. Expression of the clone in resistant Tc*Lr9* after a strong initial induction 32 hpi remained elevated for 5 days after inoculation (Fig. 8d).

An important part of the pathogen-induced response depends on calcium-mediated signaling. The expression profile of putative calcium ion binding calreticulin, encoded by a cluster of 3 clones (JG968925_JK818345_JG968926), shows pathogen-dependent temporal induction in both susceptible and resistant plants (Fig. 8e).

Expression of putative respiratory burst oxidase homolog Rboh (JG968934) is temporally induced in both tested lines, although the differences between the lines are not statistically significant (Fig. 8f). The C-terminal part of Rboh has affinity to 14-3-3 protein, which also binds H^+^-dependent ATPases. A clone with high similarity to wheat, barley and rice 14-3-3 protein (JG968969) shows transient induction 16 hpi in Thatcher and strong induction 32 hpi in resistant Tc*Lr9*. Expression of the clone remains at an elevated level up to 5 dpi (Fig. 8g).

The cumulative value of transcript rate was used to compare expression profiles of tested SSH clones and to evaluate their possible impact on plant-pathogen interaction. Among all clones the highest CVTR (770.4) was found for a clone encoding a calcium-mediated signaling protein (JG968925, Table 2, Fig. 8e). In the resistant line the value was 5.4x higher than in the susceptible Thatcher (Table 2). The CVTR for the remaining clones were low to intermediate (from 1.5 to 68.8). In resistant plants the values were from 1.5 to 5.5x higher compared with susceptible Thatcher. The highest 5.5x change of the CVTR was for the clone encoding wall-associated kinase (JG969003, Table 2, Fig. 8a).

**Table 2:** Cumulative values of transcript rates (CVTR) of selected SSH clones in susceptible Thatcher and resistant Tc*Lr9* line

Isogenic line	CVTR	
SSH clone	Thatcher	Tc*Lr9*
Wall-associated kinase JG969003 (Fig. 8a)	12.4	68.8
Wall-associated kinase JG968951 (Fig. 8b)	1.5	2.3
Receptor with leucine-rich repeat domain JG968955 (Fig. 8c)	5.0	14.3
Putative serine/threonine protein kinase JG968944 (Fig. 8d)	7.9	19.4
Calcium-mediated signaling protein JG968925 (Fig. 8e)	141.5	770.4
Rboh-like protein JG968934 (Fig. 8f)	2.2	5.6
14-3-3 protein JG968969 (Fig. 8g)	21.8	57.6

## Discussion

The SSH library was constructed using RNA isolated from leaves of isogenic lines – susceptible Thatcher and resistant Tc*Lr9* – inoculated with a single spore isolate of *P. triticina*. The time points, based on histopathological observations, represented the most important steps of plant-pathogen interactions: a high ratio of germinating spores (12 hpi), the beginning of haustorium mother cell (HMC) formation (20 hpi), a high ratio of HMCs (26 hpi), the beginning of micronecrotic reactions (32 hpi) and a strong necrotic response (36 hpi). From the original 793 cDNA clones, 247 clones were selected after differential screening as the clones potentially involved in wheat–brown rust interaction. These clones represented putative defense response genes that were differentially regulated in wheat after infection with *P. triticina*.

Gross comparison of sequence annotation with the general profile for the wheat proteome [24] indicated enrichment with sequences harboring functional domains involved in complex formation (e.g. protein, RNA and DNA binding) as well as catalytic activity (e.g. hydrolase kinase activity). Interestingly, proteins involved in ‘transferase activity’ seem to be less represented. Genes encoding proteins involved in ‘structural activity’ and ‘transducer activity’ were represented at similar levels when compared to the gross wheat Gene Ontology profile [24]. The detected profile of molecular activities provides a global overview of proteins involved in the mechanism of resistance in selected wheat lines.

Plant-pathogen interaction involves molecules/mechanisms representing different layers of plant defense, as defined by Jones and Dangle (2006) [37]. Pathogen-associated molecular patterns (PAMPs) or pathogen-derived elicitors are recognized by host pattern recognition receptors (PRR), which facilitates efficient recognition of the pathogen by the host organism. Most PRRs characterized to date are receptor-like kinases (RLKs) possessing an extracellular domain, a transmembrane domain and a kinase domain. The characterization and annotation of the transcript identified in this study indicate that they can be categorized as defense-related due to their involvement in either basal defense or resistance through an *R*-gene mediated reaction.

The postulated successive steps leading to activation of the defense response include: host recognition of the pathogen followed by triggering of a signaling cascade involving MAP kinases, calcium ion fluxes [38], generation of ROS and oxidative burst [39], enforcing physical barriers by cell wall appositions [40], the hypersensitive response (HR) and cell death. The significant numbers of transcripts identified in this study represent components of this system.

Host recognition of pathogen infection is represented by several clones. The structure of the putative receptor with a nucleotide binding (NB) and leucine-rich repeat (LRR) domain (clone JG968955) is typical for most plant resistance R proteins, and it may function in race-specific effector-triggered immunity as well as in basal resistance (reviewed previously [41, 42]). Expression of the clone, strongly and transiently induced in susceptible Thatcher and resistant Tc*Lr9* at the onset of appressoria formation (16 hpi. Fig. 8c), indicates a role in the pathogen recognition. The expression, significantly higher in Tc*Lr9*, indicates a function in rust resistance, possibly by interaction with *Lr9* in a manner similar to *Lr10*, which depends on NBS-LRR encoding the *RGA2* gene [43]. Pathogen-induced signal transduction activates a large group of protein kinases including MAP, WAK and NBS-LRR kinases. The following clones of the cDNA subtracted library represent Ser/Thr protein kinases: JG968978, JK818355, JK818407, JG968988, JG968943_JG968944, mitogen-associated protein kinases: JK818367, JG968966, receptor protein kinases with the NB-ARC domain JG968955 and wall-associated kinases: JG968951, JG968933_JK818362 and JG968950_JG969003. The putative serine/threonine protein kinase (clone JG968944) representing genes possibly involved in signaling is significantly higher upregulated in resistant Tc*Lr9* than susceptible Thatcher (Fig. 8d). It is noteworthy that induction of the gene remains high 5 dpi despite no signs of pathogen structures on the leaves.

Five ESTs identified in the SSH library represent three transcripts (JG968933_JK818362, JG968951, JG969003_JG968950) of putative WAKs. The common structural features of WAK proteins are the cytoplasmically localized Ser/Thr kinase domain and extracellularly localized epidermal growth factor (EGF) domains. The WAK genes exist as large gene families. There are 26 *WAK* and *WAK-like* genes in *Arabidopsis* and 125 in rice [44]. It has been postulated that WAKs’ ability to bind different types of pectins [45] and oligogalacturonides (OGs) [46] and subsequent activation of kinase domain facilitates the biological functions of the genes – i.e. regulation of cell elongation, morphogenesis, and plant immune responses [46, 47, 48].

There are increasing numbers of reports indicating direct involvement of wall-associated kinases in signaling in the plant response to pathogen infection. In rice *OsWAK1* was found to be induced by *Magnaporthe oryzae* and its functional analysis confirmed the crucial role of the gene in resistance against rice blast [49]. Molecules termed microbe-associated molecular patterns (MAMPs) and damage-induced molecular patterns (DAMPs) function as inducers of diverse layers of plant immune response: calcium-dependent signaling, production of reactive oxygen species (ROS) and induction of mitogen-activated protein kinase (MAPK) cascades [50]. The wall-associated kinase-encoding *SIWAK1* was identified as a gene induced by MAMPs and functioning in tomato resistance against *Pseudomonas syringae* [51]. According to the authors, SIWAK1, a receptor of infection-generated OGs, triggers pathogen-induced signaling. Map-based cloning of *qHSR1* led to identification of the maize gene *ZmWAK* encoding wall-associated kinase and conferring resistance to the soil-borne fungal pathogen *Sporisorium reilianum* [52]. It is worth noting that the gene confers quantitative resistance, which is very important in plant breeding because it is usually more durable then qualitative resistance conferred by major resistance genes [53].

The 3 wheat putative *WAKs* identified in the SSH library (JG968933, JG969003 and JG968951) are strongly induced by pathogen infection (Fig. 8a, b). Moreover, the expression profiles of *TaWAK* (JG968933) correlated with the resistance level, differentiating the three groups of genotypes: susceptible Thatcher from medium resistant (Tc*Lr24*, Tc*Lr25*) and highly resistant (Tc*Lr9* and Tc*Lr26*) lines (Fig. 7). The high correlation coefficient between the *TaWAK* (JG968933) CVTRs and the level of the resistance is a strong indication for the important role in wheat resistance against brown rust. The function of the gene is currently under a detailed functional analysis. The presence of a Ca^2+^-binding domain in TaWAK (JG968933, Fig. 7) and the ability to bind OGs [46] indicate that this receptor kinase after perceiving pathogen-derived signals (i.e. elicitors/OGs) can initiate oxidative burst through Ca^2+^ signaling. The feature provides the link with the following set of EST clones. Calcium ions act as a crucial second messenger in passing pathogen-induced signals from receptors (such as receptor kinases) to target molecules and activate defense reactions including HR [38, 54, 55]. In the subtractive library calcium-binding molecules are represented by a relatively large group of ESTs encoding calmodulin-binding proteins/calcium-binding proteins and calcium-transporting ATPases including those with H^+^/Ca^2+^ antiporter activity to restore the initially low concentration of Ca^2+^ ions in the cytoplasm. The following clones represent pathogen-regulated calcium transporting ATPases: JK818321_JK818338 and JG968975_JG968986, JG968929, JG968930_JG968931, JG969002_JG968938_JG968937.

Our previous results indicated that incompatible wheat-brown rust interaction was associated with activation of the two enzymatic systems peroxidases and NADPH oxidases known as respiratory burst oxidase homologs (Rboh) and accumulation of ROS in stomata and mesophyll cells around the infection site [10]. Rboh are plasma membrane proteins directly controlled by calcium ions through N-terminal EF-hand calcium-binding motifs [56]. Cytosolic Ca^2+^ spiking, which precedes pathogen-induced defense responses, is a likely factor initiating Rboh activity and oxidative burst [57]. Expression of the *Rboh-like* encoding gene (JG968934) correlates very well with the pattern of oxidative burst in the susceptible Thatcher and the resistant Tc*Lr9* line [10, 11].

Biphasic expression of the calcium-mediated signaling clone (JG968925, Fig. 8e) and H_2_O_2_ generating Rboh (JG968934, Fig. 8f) is similar to the pattern of cytosolic calcium influx detected in *Arabidopsis* plants challenged by *P. syringa*e [58]. This agrees with a concept postulating that close localization of the Rboh and the Ca^2+^ channels facilitates close control of oxidative burst in sites adjacent to pathogen infection [55]. It is also compatible with the observation of calcium-dependent activation of Rboh (NADPH oxidase) and H_2_O_2_ generation in OG-treated *N. plumbaginifilia* [59]. In our system the biphasic pattern of processes observed in wheat-rust interaction is evident: the first peak of the expression (Fig. 8a-g) and the accumulation of ROS in stomata correlates well with formation of appressoria on stomata [10]. The second expression signal, detected only in the Tc*Lr9* resistant line, correlates with oxidative burst in mesophyll cells and micronecrotic/hypersensitive (HR) reactions [10]. It is worth noting that one of the two identified WAKs (JG968933) with a possible role in calcium signaling (*calcium ion binding* identified by GO analysis) showed strong induction during the incompatible wheat-rust interaction (Fig. 7). The results indicate that the identified TaWAK might function as the receptor of OGs (which are possible components of PAMPs) and also as the calcium signaling component.

Phosphatidic acid (PA) acts as a functional molecule and secondary messenger in pathogen-induced phospholipid signaling [60]. The PA-dependent pathway intertwines with calcium signaling. It activates the MAP kinase cascade and triggers oxidative burst by recruiting functional NADPH-complex (Rboh) at the plasma membrane [60]. The components of this pathway are represented by SSH clones encoding phospholipase D (JG968992, JK818323_JK818358) and phosphatidylcholine cytidylyltransferase (JG968942, JG968943_JG968944). The Rac/Rop GTPases (small GTPases) constitute part of a large and divergent group of monomeric GTP-binding proteins (mG-proteins) and act as important components of plant immunity. They form protein complexes with chaperones Hsp90 and Hsp70 [61] and function as molecular switches interconnecting ROS generating systems, calcium signaling and cell wall enforcement [62]. Rice Rac GTPase *OsRac1* was found to be involved in ROS generation by directly interacting in a calcium-dependent manner with the N-terminal region of Rboh (NADPH oxidase) [63]. Monomeric GTP-binding proteins are represented in SSH library by the following clones: JG969001, JK818377, JG969000, JG968940, JG969002_JG968938_JG968937, JG968995_JG968994_JG968993, JG968948_JG968961, JK818408 and JG968940.

Chaperones play an important role in plant resistance by maintaining cell homeostasis during infection. Many chaperones have been identified as heat shock proteins (Hsp), and some of them, such as Hsp60, Hsp70 and Hsp90, were defined as pathogen-related (PR) proteins. It has been found that wheat resistance to *Puccinia graminis* conferred by the *TaRLK-R* gene [64] and wheat resistance to *P. triticina* conferred by *Lr21* [65] are functionally dependent on Hsp90s [66]. The following identified cDNA clones have heat shock protein signatures with putative chaperone functionalities: JK818328, JK818388, JG968981 and JK818410.

Another group of components of the immune network constitutes 14-3-3 proteins. They act as intracellular signal molecules coordinating different signaling pathways. They participate in regulation of ROS production by direct interaction with Rboh (NADPH oxidase) and coregulation by small GTPase of the Rac family [67]. By regulation of pathogen-induced oxidative burst the 14-3-3 proteins also positively regulate programmed cell death (PCD)/HR [68]. Expression of 14-3-3 encoding genes was reported during incompatible barley-powdery mildew [69] and soybean–*Pseudomonas syringae* [70] interactions. The 14-3-3 proteins are encoded by JG968970 and JG968969 clones. The expression profile of the 14-3-3 encoding clone (JG968969) with induction during the first phase of wheat-rust interaction and strong up-regulation only in resistant Tc*Lr9* (Fig. 8g) confirms its involvement in rust resistance.

All considered, the subtractive library contains ESTs with a demonstrated or highly probable role in plant-pathogen interaction and represents wheat transcripts differentially regulated during plant-pathogen interaction, which participate in the most important pathways involved in the plant response to pathogen infection. Further detailed analysis of the relevant genes should provide new information about mechanisms of wheat resistance to rust infection and supply novel candidates for modern plant breeding using molecular techniques.

## Conclusions

The SSH library represents transcripts regulated by pathogen infection during compatible and incompatible interactions of susceptible Thatcher and resistant *Lr9* with *Puccinia triticina*.

Annotation of selected clones confirmed their putative roles in the successive steps of plant-pathogen interactions. This included pathogen recognition, pathogen-induced signaling and resistance processes such as cell wall enforcement, oxidative burst and micronecrotic reactions.

Among the clones strongly up-regulated during incompatible interaction were the transcripts of wall-associated kinases, which due to their transmembrane localization might function as signal transducers, the transcripts involved in calcium-mediated signaling and the transcripts encoding Rboh-like proteins with possible roles in oxidative burst and micronecrotic reactions.

## Additional files

Additional file 1 : Table S4. Infection types of selected isogenic wheat lines inoculated with single spore isolate of *Puccinia triticina* (brown rust) used in this study. Infection type scale: 0 – no uredinia or other macroscopic signs of infection, 0; − no uredinia, but hypersensitive necrotic or chlorotic flecks of varying size present, 1 – small uredinia often surrounded by necrosis, 2 – small to medium-sized uredinia often surrounded by chlorosis or necrosis, 3 – medium-sized uredinia associated with chlorosis or rarely necrosis, 4 – large uredinia without chlorosis or necrosis. (DOCX 19 kb) Additional file 2: Table S1. Primers and reaction conditions used for PCR. (DOCX 18 kb) Additional file 3: Table S2. Expression sequence tags of wheat (line Tc*Lr9*) after inoculation with brown rust (*Puccinia triticina*) deposited in GenBank http://www.ncbi.nlm.nih.gov/Genbank. (DOCX 18 kb) Additional file 4: Table S3. Gross functional annotation of EST tags. (XLS 95 kb)

## Abbreviations

CVTR: Cumulative value of transcript rate
dpi: Days post inoculation
GO: Gene ontology
HMC: Haustorium mother cell
hpi: Hours post inoculation
PAMP: Pathogen-associated molecular patterns
PRR: Pattern recognition receptors
qPCR: Quantitative PCR
Rboh: Respiratory burst oxidase homologue
RT-PCR: Reverse transcription polymerase chain reaction
SSH: Suppression subtractive hybridization
WAK: Wall-associated kinase
