# Supplementary material for: Pathogen-regulated genes in wheat isogenic lines differing in resistance to brown rust Puccinia triticina
Source: BMC Genomics. 2015 Oct 5;16:742. doi: 10.1186/s12864-015-1932-3 (PMC4595183; doi:10.1186/s12864-015-1932-3)
Supplement: Additional file 2: Table S1. — Primers and reaction conditions used for PCR. (DOCX 18 kb) [file 12864_2015_1932_MOESM1_ESM.docx]

Additional file 1

**Table S4.** Infection types of selected isogenic wheat lines inoculated with single spore isolate of *Puccinia* *triticina* (brown rust) used in this study.

Infection type scale: 0 – no uredinia or other macroscopic signs of infection, 0; – no uredinia, but hypersensitive necrotic or chlorotic flecks of varying size present, 1 – small uredinia often surrounded by necrosis, 2 – small to medium-sized uredinia often surrounded by chlorosis or necrosis, 3 – medium-sized uredinia associated with chlorosis or rarely necrosis, 4 – large uredinia without chlorosis or necrosis.

| ***Lr* gene** | **Wheat line description** | **Line number** | **Infection type** |
| --- | --- | --- | --- |
| *Lr 1* | Tc*6/Centenario | RL6003 | 4 |
| *Lr 2a* | Tc*6/Webster | RL6016 | 3 |
| *Lr 2b* | Tc*6/Carina | RL6019 | 3 |
| *Lr 2c* | Tc*6/Loros | RL6025 | 3 |
| *Lr 3* | Tc*6/Democrat | RL6002 | 1 |
| *Lr 3bg* | Báge/Tc*8 | RL6042 | 0; |
| *Lr 3ka* | Tc*6/Aniversario | RL6007 | 1 |
| *Lr 9* | Transfer/Tc*6 Aegilops.umbellulata | RL6010 | 0 |
| *Lr 10* | Tc*6/Exchange | RL6004 | 4 |
| *Lr 11* | Tc*6/Hussar | RL6053 | 4 |
| *Lr 12* | Exchange/Tc*6 | RL6011 | 4 |
| *Lr 13* | Tc*7/Frontana | RL4031 | 3 |
| *Lr 14a* | Selkirk/Tc*6 | RL6013 | 4 |
| *Lr 14b* | Tc*6/Maria Escobar | RL6006 | 4 |
| *Lr 15* | Tc*6/Kenya W1483 | RL6052 | 2 |
| *Lr 16* | Tc*6/Exchange | RL6005 | 2 |
| *Lr 17* | Klein Lucero/Tc*6 | RL6008 | 1 |
| *Lr 18* | Tc*7/South Afrika 43 | RL6009 | 4 |
| *Lr 19* | Tc*7Transloc.4-*Agropyron elongatum* | RL6040 | 0; |
| *Lr 20* | Tc*6/Timmo | RL6092 | 1 |
| *Lr 21* | Tc*6/RL5406 *Ae. squarrosa* v. *meyeri* | RL6043 | 3 |
| *Lr 22* | Tc*6/RL5404 *Ae.squarrosa* v. *strangulata* | RL6044 | 4 |
| *Lr 23* | Lee FL310/Tc*6 | RL6012 | 1 |
| *Lr 24* | Tc*6/Agent (*Agropyron elongatum*) | RL6064 | 1 |
| *Lr 25* | Tc*6/Transec (*Secale cereale*) | RL6084 | 2 |
| *Lr 26* | Tc*6/ST-1.25 | RL6078 | 0 |
| *Lr 28* | Tc*6/C77.1 (*Aegilops speltoides*) | RL6079 | 1 |
| *Lr 29* | Tc*6/CS7D/Ag#11 (*A. elongatum*) | RL6080 | 1 |
| *Lr 30* | Tc*6/Terenzio | RL6049 | 2 |
| *Lr 32* | Tc*6/3/ *Aegilops squarrosa* | RL6086 | 4 |
| *Lr 33* | Tc*6/PI 58548-1 (1+gene) | RL6057 | 4 |
| *Lr 34* | Tc*6/PI 58548-2 (2+gene) | RL6058 | 4 |
| *Lr 35* | Tc*6/RL 5711 *T. speltoides* | RL6083 | 4 |
| *Lr 37* | Tc*8/VPMI *T.ventricosum* | RL6081 | 4 |
| *Lr 38* | Tc*6/T7 | RL6097 | 4 |
| *Lr 38* | Tc*6/TMR-514-12-24 | RL6137 | 1 |
| *Lr 44* | Tc*6/*T.spelta* 7831 | RL6147 | 2 |
| *Lr B* | Tc*6/Carina | RL6051 | 4 |
| *Lr B* | Tc*6/PI 268316 | RL6061 | 4 |
| *Lr W* | Tc*6/V336 | RL6107 | 3 |
| - | Thatcher |  | 4 |
