# Supplementary material for: Pathogen-regulated genes in wheat isogenic lines differing in resistance to brown rust Puccinia triticina
Source: BMC Genomics. 2015 Oct 5;16:742. doi: 10.1186/s12864-015-1932-3 (PMC4595183; doi:10.1186/s12864-015-1932-3)
Supplement: Additional file 3: Table S2. — Expression sequence tags of wheat (line TcLr9) after inoculation with brown rust (Puccinia triticina) deposited in GenBank http://www.ncbi.nlm.nih.gov/Genbank. (DOCX 18 kb) [file 12864_2015_1932_MOESM2_ESM.docx]

Additional file 2

Table S1. The primers and the reaction conditions used for PCR.

| **Gene** | **Primers** | **Conditions** |
| --- | --- | --- |
| Universal primers | T7: AATACGACTCACTATAGGG  SP6: ATTTAGGTGACACTATAG |  |
| BD PCR-Select cDNA Subtractive Hybridization Kit | Nested PCR Primer1: TCGAGCGGCCGCCCGGGCAGGT  Nested PCR Primer2R: AGCGTGGTCGCGGCCGAGGT |  |
| Wheat *pinB*  DQ363913 [20] | PB3: GAGCCTCAACCCATCTATTCATC  PB4: CAAGGGTGATTTTATTCATAG |  |
| Wheat 18S rRNA  M82356 [37] | Forward: GTGACGGGTGACGGAGAATT  Reverse: GACACTAATGCGCCCGGTAT | 95 ºC 15 min, 35 cycles (95 ºC 25 s, 58ºC 25 s, 72 ºC 25 s) |
| Calcium-mediated signaling  JG968925 | RI-37_F: GGCAAGATGTCCCAATCAGT  RI-37_R: GCCCGACGCATCTTATAGTT | 95 ºC 15 min, 37 cycles (95 ºC 25 s, 60 ºC 25 s, 72 ºC 25 s) |
| Wall-associated kinase  JG969003 | FII-42_F: ATTGATGGCCTGTTCTCACC  FII-42_R: TTTAATCTCGATGCCCTTGG | 95 ºC 15 min, 45 cycles (95 ºC 25 s, 61 ºC 25 s, 72ºC 25 s) |
| Wall-associated kinase  JG968951 | FII-52_F: CCTCGCTCGTCACACAAATA  FII-52_R2: TCTACCCTTTTGGCTTGCAC | 95 ºC 15 min, 45 cycles (95 ºC 25 s, 57 ºC 25 s, 72 ºC 25 s) |
| Serine/threonine protein kinase  JG968944 | RII-26_F: CGATTGGGATACACGTTTCA  RII-26_R: CAAAGTCCGCAATCTTAGGC | 95 ºC 15 min, 45 cycles (95 ºC 25 s, 60 ºC 25 s, 72 ºC 25 s) |
| Receptor with LRR domain  JG968955 | RII-62_F: TCTCCACACTGCTCCTCATCT  RII-62_R: CCGAGGACTTCAGCTTCTTG | 95 ºC 15 min, 45 cycles (95 ºC 25 s, 60 ºC 25 s, 72 ºC 25 s) |
| 14-3-3 protein  JG968969 | FII-126_F: GGACAGCTTAGGCGAGGAAT  FII-126_R: CAACGTCATCCTCATTGGTG | 95 ºC 15 min, 40 cycles (95 ºC 25 s, 60 ºC 25 s, 72 ºC 25 s) |
| Rboh-like gene  JG968934 | RI-74_F: TATTACTGTCGTGCCGGTGA  RI-74_R: GTGGCCTTCCCTATTGTCCT | 95 ºC 15 min, 45 cycles (95 ºC 25 s, 60 ºC 25 s, 72 ºC 25 s) |
| Wall-associated kinase (*TaWAK*)  JG968933 | Kinr_5nR: CAAAACCACCACTTCCAATAATGTTGC  Ri77ant: GTTGGCGGACCTACATTGGAAAAGCTTA | 95 ºC 15 min, 47 cycles (95 ºC 25 s, 62 ºC 25 s, 72 ºC 25 s) |
