# Supplementary material for: Pathogen-regulated genes in wheat isogenic lines differing in resistance to brown rust Puccinia triticina
Source: BMC Genomics. 2015 Oct 5;16:742. doi: 10.1186/s12864-015-1932-3 (PMC4595183; doi:10.1186/s12864-015-1932-3)
Supplement: Additional file 4: Table S3. — Gross functional annotation of EST tags. (XLS 95 kb) [file 12864_2015_1932_MOESM3_ESM.docx]

**Additional file 3**

**Table S2.** Expression sequence tags of wheat line Tc*Lr9* after inoculation with brown rust *Puccinia triticina* deposited in the GenBank <http://www.ncbi.nlm.nih.gov/Genbank>

| **dbEST_Id** | **User_Id** | **GenBank_Accn** | **dbEST_Id** | **User_Id** | **GenBank_Accn** |
| --- | --- | --- | --- | --- | --- |
| 74112561 | FI-5 | **JG968919** | 74112604 | FII-98 | **JG968962** |
| 74112562 | FI-7 | **JG968920** | 74112605 | FII-99 | **JG968963** |
| 74112563 | FI-8 | **JG968921** | 74112606 | RII-101 | **JG968964** |
| 74112564 | FI-13 | **JG968922** | 74112607 | FII-102 | **JG968965** |
| 74112565 | FI-14 | **JG968923** | 74112608 | RII-119 | **JG968966** |
| 74112566 | FI-16 | **JG968924** | 74112609 | FII-121 | **JG968967** |
| 74112567 | RI-37 | **JG968925** | 74112610 | FII-125 | **JG968968** |
| 74112568 | RI-53 | **JG968926** | 74112611 | FII-126 | **JG968969** |
| 74112569 | FI-22 | **JG968927** | 74112612 | FII-122 | **JG968970** |
| 74112570 | FI-24 | **JG968928** | 74112613 | FII-123 | **JG968971** |
| 74112571 | FI-31 | **JG968929** | 74112614 | RII-124 | **JG968972** |
| 74112572 | FI-32 | **JG968930** | 74112615 | FII-124 | **JG968973** |
| 74112573 | FI-33 | **JG968931** | 74112616 | FIII-13 | **JG968974** |
| 74112574 | RI-76 | **JG968932** | 74112617 | FIII-14 | **JG968975** |
| 74112575 | RI-77 | **JG968933** | 74112618 | FIII-15 | **JG968976** |
| 74112576 | RI-74 | **JG968934** | 74112619 | FIII-27 | **JG968977** |
| 74112577 | RI-80 | **JG968935** | 74112620 | FIII-31 | **JG968978** |
| 74112578 | RI-89 | **JG968936** | 74112621 | FIII-43 | **JG968979** |
| 74112579 | RI-114 | **JG968937** | 74112622 | FIII-47 | **JG968980** |
| 74112580 | RI-143 | **JG968938** | 74112623 | FIII-54 | **JG968981** |
| 74112581 | FII-2 | **JG968939** | 74112624 | FIII-56 | **JG968982** |
| 74112582 | FII-4 | **JG968940** | 74112625 | FIII-104 | **JG968983** |
| 74112583 | FII-10 | **JG968941** | 74112626 | FIII-145 | **JG968984** |
| 74112584 | FII-11 | **JG968942** | 74112627 | FIII-117 | **JG968985** |
| 74112585 | RII-24 | **JG968943** | 74112628 | FIII-118 | **JG968986** |
| 74112586 | RII-26 | **JG968944** | 74112629 | FIII-121 | **JG968987** |
| 74112587 | FII-25 | **JG968945** | 74112630 | RIII-121 | **JG968988** |
| 74112588 | FII-26 | **JG968946** | 74112631 | FIII-124 | **JG968989** |
| 74112589 | FII-27 | **JG968947** | 74112632 | FIII-128 | **JG968990** |
| 74112590 | FII-29 | **JG968948** | 74112633 | FIII-130 | **JG968991** |
| 74112591 | FII-35 | **JG968949** | 74112634 | FIII-132 | **JG968992** |
| 74112592 | FII-44 | **JG968950** | 74112635 | FIII-136 | **JG968993** |
| 74112593 | FII-52 | **JG968951** | 74112636 | FIII-137 | **JG968994** |
| 74112594 | FII-56 | **JG968952** | 74112637 | FIII-150 | **JG968995** |
| 74112595 | FII-74 | **JG968953** | 74112638 | FIII-139 | **JG968996** |
| 74112596 | FII-75 | **JG968954** | 74112639 | FIII-146 | **JG968997** |
| 74112597 | RII-62 | **JG968955** | 74112640 | FIII-153 | **JG968998** |
| 74112598 | FII-77 | **JG968956** | 74112641 | FIII-147 | **JG968999** |
| 74112599 | FII-78 | **JG968957** | 74112642 | FIII-149 | **JG969000** |
| 74112600 | FII-81 | **JG968958** | 74112643 | FIII-155 | **JG969001** |
| 74112601 | FII-82 | **JG968959** | 74112644 | RI-150 | **JG969002** |
| 74112602 | FII-92 | **JG968960** | 74112645 | FII-42 | **JG969003** |
| 74112603 | FII-97 | **JG968961** | 74112646 | FII-164 | **JG969004** |
